# Phubbing, Being Phubbed, and Interpersonal Communication Quality Across Generations X, Y, and Z: Evidence of a Generational Asymmetry

**DOI:** 10.3390/bs16091536

**Published:** 2026-08-31

**Authors:** Samet Candar, Fatma Geçikli

**Affiliations:** Department of Public Relations and Publicity, Faculty of Communication, Atatürk University, Erzurum 25240, Türkiye; fgecikli@atauni.edu.tr

**Keywords:** phubbing, being phubbed, interpersonal communication quality, generational cohorts, digital natives, normalization

## Abstract

Phubbing, the act of snubbing a conversational partner in favor of a mobile phone, has become a routine feature of face-to-face interaction. This study tested seven directional hypotheses regarding general phubbing, being phubbed, and interpersonal communication quality across Generations X, Y, and Z, drawing on cohort theory and the digital-native framework. Data were collected via face-to-face questionnaires from 916 students and academic and administrative staff at a large Turkish public university using convenience sampling, and were analyzed with ANOVA, regression, and confirmatory factor analysis. General phubbing and being phubbed were measured with the Turkish-adapted Generic Scale of Phubbing and Generic Scale of Being Phubbed; communication quality was measured with the Interpersonal Communication Scale. General phubbing increased stepwise from Generation X to Z and was significantly and negatively associated with communication quality, as hypothesized. Being phubbed did not differ by generation, consistent with a normalization account, and showed a small, unhypothesized positive association with communication quality. Neither relationship was moderated by generational membership. Generation Z reported the lowest communication quality of the three cohorts, a result robust to sensitivity checks. Measurement limitations and implications for generational research on digital communication are discussed.

## 1. Introduction

Phubbing, a portmanteau of “phone” and “snubbing,” refers to the act of attending to a mobile phone during a face-to-face social interaction at the expense of the conversational partner (Chotpitayasunondh & Douglas, 2016). The construct is now conventionally split into two related but distinct components: general phubbing, an individual’s own tendency to divert attention toward a phone during interaction, and being phubbed, the experience of having a conversational partner do the same (Chotpitayasunondh & Douglas, 2018a). This distinction matters because the antecedents, prevalence, and consequences of phubbing someone and of being phubbed need not coincide, and the present study treats them as separate constructs throughout.

A substantial body of work has linked general phubbing to reduced interaction quality. Even the passive co-presence of a phone during a conversation, without active use, has been shown to lower the reported quality of that conversation (Dwyer et al., 2018). Individuals who phub their partners report more relational strain (Roberts & David, 2016), and both the antecedents and interpersonal costs of phubbing extend into everyday friendships as well as romantic relationships (Sun & Samp, 2022). In the Turkish context specifically, phubbing has been associated with lower relationship satisfaction despite being widely tolerated as an unremarkable behavior (Çizmeci, 2017). Chotpitayasunondh and Douglas (2018b) further showed experimentally that phubbing reduces perceived communication quality, relational satisfaction, and a sense of belonging among those on the receiving end. These relational costs are not confined to romantic partners: perceived partner phubbing predicts lower relationship quality even when the phubbing partner’s own reported behavior does not (Carnelley et al., 2023), and supervisor phubbing in the workplace undermines subordinates’ trust, job satisfaction, and performance (Roberts & David, 2020).

Beyond its consequences, research on general phubbing has also examined who engages in it and how widespread it is. Trait boredom, for instance, predicts phubbing frequency (Al-Saggaf et al., 2019), and off-task smartphone use of this kind carries measurable costs in academic settings (Abramova et al., 2017). Aagaard (2020) characterizes habitual phubbing as a form of “digital akrasia,” a felt loss of control over attention that persists despite users’ disapproval of the behavior; prevalence estimates among students in comparative European samples confirm that the behavior is widespread rather than exceptional (Barbed-Castrejón et al., 2024). At the same time, recent cross-cultural work shows that the interpretation of co-present phone use as phubbing varies across national contexts, so phubbing should not be treated as a culturally uniform category (Büttner et al., 2025).

Two theoretical accounts explain why general phubbing should compromise communication quality. Media Richness Theory holds that face-to-face communication constitutes the richest available channel because it permits immediate feedback, multiple non-verbal cues, and personal focus; anything that fragments attention during this channel reduces its effective richness (Daft & Lengel, 1986). The interaction involvement framework similarly treats cognitive engagement, responsiveness, and perceptiveness toward a partner as core components of communicative competence, all of which are directly undermined when attention shifts to a device (Cegala, 1981). Within intercultural and negotiation settings, the quality of a communication experience has likewise been framed as a joint product of participants’ mutual engagement and affective connection during the exchange (Liu et al., 2010), a framing that phubbing directly threatens by removing one party’s engagement from the interaction.

Generational cohort theory offers the second theoretical anchor for this study. Mannheim’s (1952) classical account of generations holds that individuals who pass through the same formative period of life together, and who are consequently exposed to the same historical and technological conditions during that period, tend to develop a shared, distinctive orientation toward the social world that outlasts the period itself. Applied to digital technology, this logic underlies the widely used, if contested, distinction between digital natives, who grow up immersed in digital tools from early childhood, and digital immigrants, who adopt them later in life and consequently relate to them differently (Prensky, 2001). Contemporary demographic classifications operationalize this shared-formative-period logic into three cohorts relevant here: Generation X, Generation Y (Millennials), and Generation Z (Dimock, 2019; Strauss & Howe, 1991). Because the hypotheses were formulated in terms of cohort membership, generation rather than chronological age was used as the primary comparative variable; age remains available in the dataset and is reported descriptively within each cohort. Generational membership is used here as a birth-year-based analytical grouping derived from cohort theory, not as a direct measure of individual technological experience. The digital-native/immigrant dichotomy, however, has been criticized as an oversimplified and empirically under-supported binary that risks treating a genuinely heterogeneous cohort as homogeneous (Bayne & Ross, 2007; Bennett et al., 2008; Brown & Czerniewicz, 2010). Such homogenization can become a mechanism of intergenerational stereotyping when cohort-level tendencies are treated as fixed attributes of individual members. The labels may obscure within-generation variation and encourage assumptions about individuals’ technological competence, communication preferences, or interpersonal abilities on the basis of cohort membership rather than observed characteristics or behavior. For example, individuals categorized as Generation Z may be presumed to be uniformly technologically proficient but comparatively weak in face-to-face communication, whereas members of older cohorts may be presumed to be less digitally competent simply because they are positioned as “digital immigrants.” In this way, an analytical distinction intended to describe formative technological contexts can become an evaluative boundary that produces unjustified intergenerational stereotypes. Accordingly, the present study treats generational membership as an analytical classification rather than as a deterministic indicator of technological exposure, competence, or behavior, and interprets generational comparisons accordingly. Broader generational-values research reinforces the expectation that cohorts differ systematically in self-focus and communication style as a function of the technological and cultural conditions of their formative years (Twenge, 2006).

A further theoretical implication follows from treating phubbing as an increasingly normalized social practice. Chotpitayasunondh and Douglas (2016) argue that phubbing has become embedded in everyday social norms to the point that its occurrence is no longer strongly contingent on any single demographic characteristic of the observer, but rather reflects the behavior’s pervasiveness across the social environment as a whole. If exposure to others’ phone use has indeed become a broadly shared feature of the contemporary communication environment, generational differences should be markedly smaller, or absent, for being phubbed than for engaging in phubbing oneself, even though the latter may still vary across cohorts because it is an individually enacted behavior rather than exposure to the surrounding social environment. This asymmetry follows from what normalization implies for each construct rather than being an ad hoc distinction: a broadly shared norm of phone-centered interaction changes what a person is exposed to from others regardless of that person’s own cohort, because it describes a property of the surrounding social environment rather than of the individual. Engaging in phubbing oneself, by contrast, remains an individually enacted behavior whose frequency may differ across birth-year cohorts rather than being determined solely by awareness that the behavior is common; knowing that phubbing is normative does not by itself equalize how often a person personally reaches for a phone during interaction. Normalization and cohort-related differences are therefore expected to operate on different sides of the same interaction, which is the basis for treating being phubbed and general phubbing as subject to different generational predictions rather than a single generation-uniform one. This normalization account, together with a broader critical literature on the social consequences of mobile phones (Vanden Abeele, 2020), directly motivates several of the hypotheses below and offers a specific alternative to a purely additive, generation-uniform prediction across both constructs. Despite the breadth of existing phubbing research (Capilla Garrido et al., 2021), comparatively few studies have tested general phubbing, being phubbed, and communication quality within a single model that also evaluates whether generational membership moderates these associations, particularly outside Western contexts.

**Figure 1:**
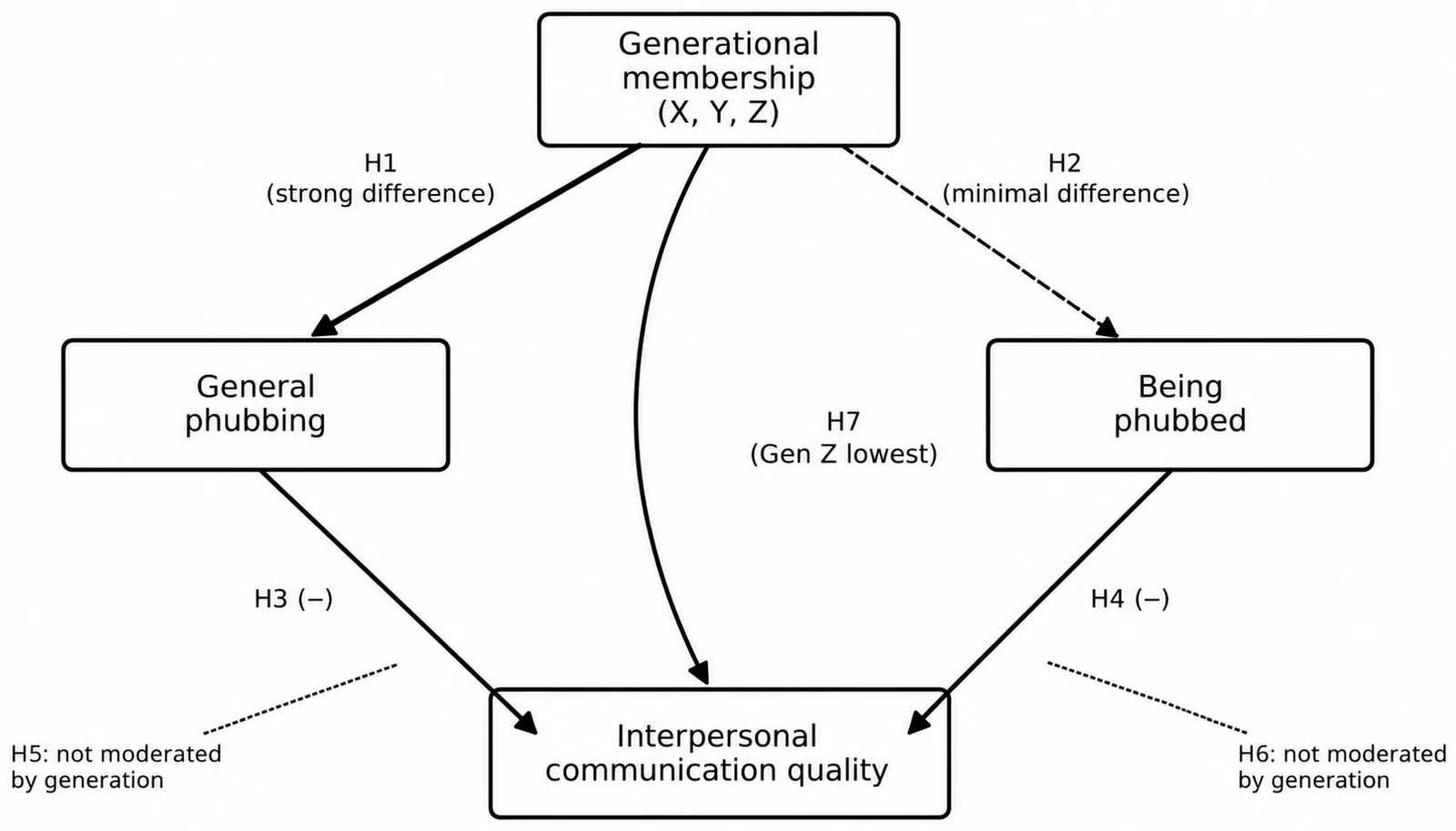
Proposed research model. Generational membership (X, Y, Z) is hypothesized to differentiate general phubbing (H1) more strongly than being phubbed (H2), and the associations of general phubbing (H3) and being phubbed (H4) with interpersonal communication quality are hypothesized to be generation-invariant (H5, H6), while communication quality itself is hypothesized to be lowest in Generation Z (H7).

Building on this framework, the present study tested the following directional hypotheses and used the research model shown in Figure 1.

**H1.**  *General phubbing will increase stepwise from Generation X to Generation Y to Generation Z*.

**H2.**  *Generational differences will be substantially smaller for being phubbed than for general phubbing, reflecting the normalization of exposure to others’ phone use as a broadly shared feature of the contemporary communication environment rather than a generation-specific tendency*.

**H3.**  *General phubbing will be significantly and negatively associated with interpersonal communication quality*.

**H4.**  *Being phubbed will be significantly and negatively associated with interpersonal communication quality*.

**H5.**  *The negative association between general phubbing and communication quality will not be moderated by generational membership, consistent with a generation-invariant pattern despite differing behavioral base rates across generations (H1)*.

**H6.**  *The association between being phubbed and communication quality will likewise not be moderated by generational membership, mirroring the normalization logic behind H2*.

**H7.**  *Generation Z will report significantly lower interpersonal communication quality than Generations X and Y*.

## 2. Materials and Methods

### 2.1. Research Design

This study used a quantitative, cross-sectional survey design. Data were collected through a structured, self-administered questionnaire delivered face-to-face on a university campus.

### 2.2. Population, Sampling, and Generational Classification

The population consisted of students and academic and administrative staff at a large Turkish public university (Atatürk University, Erzurum) during the 2024–2025 academic year, for a total accessible population of 255,393 individuals. A nonprobability convenience sampling procedure was used because reaching the full population was not feasible. As a conventional benchmark for sample adequacy, standard tables indicate that for populations exceeding 100,000, with a sampling error of d = 0.05 and *p* = 0.50, a minimum of 384 cases is typically recommended (Yazıcıoğlu & Erdoğan, 2004); the achieved sample of 916 valid responses exceeds this benchmark. This calculation is reported only as an adequacy heuristic and does not imply that the convenience sample has the representativeness properties of a probability sample; generalizability remains constrained by the sampling method itself, as noted in Section 4.6. Employment or student status was not recorded as a demographic variable in the questionnaire, and no recruitment or administration records were retained from which this information could be reconstructed retrospectively. Consequently, the occupational composition of each generational cohort cannot be verified, and this constraint is addressed directly as a major internal-validity limitation in Section 4.6.

Generational membership was assigned from reported birth year using the following operational boundaries, informed by widely used international generational classifications (Dimock, 2019; Strauss & Howe, 1991): Generation X (born 1965–1980), Generation Y (born 1981–1996), and Generation Z (born 1997–2012). The birth-year boundaries used in this study were adopted as analytical conventions to facilitate comparability with widely used generational classifications, rather than as objectively discrete biological or psychological categories. Exact cohort boundaries vary across sources and are not universally agreed upon (Dimock, 2019, 2023; Parry & Urwin, 2021). This operationalization was retained to preserve comparability with the generational framework underlying the hypotheses; however, its application in Türkiye is treated as an analytical choice rather than as evidence that the same cohort boundaries have identical sociocultural meaning across contexts. Turkish scholarship has also documented variation in the birth-year ranges used to define generational cohorts (Erden Ayhün, 2013), while comparative evidence from the United States and Türkiye indicates that U.S.-originated generational classifications cannot be assumed to generalize directly to the Turkish context (Gurbuz & Aytekin, 2020). Accordingly, the categories used here are treated as operational birth-year groupings for analytical comparison rather than as culturally validated or internally homogeneous Turkish generation types. Generational membership is therefore not interpreted as a direct measure of an individual’s digital exposure, smartphone use, technological competence, or digital dependence. The nominal upper boundary assigned to Generation Z does not affect participant classification in the present sample, since all respondents were at least 17 years old at the time of data collection.

Although the operational Generation X range was 1965–1980, 14 participants (11.1% of the 126 Generation X respondents) were aged between 61 and 66 years and were therefore born before 1965. Because Generation X was the oldest cohort included in the study, these participants were retained within Generation X rather than excluded or assigned to a separate cohort. To evaluate whether this pragmatic classification affected the results, both hypothesis tests involving generational comparisons (H1 and H7) were re-estimated after excluding these 14 cases; the results were materially unchanged (Section 3.5), and the full sample was therefore retained in the primary analyses reported below.

### 2.3. Sample Characteristics

The sample comprised 427 men (46.6%) and 489 women (53.4%); 240 participants (26.2%) were married and 676 (73.8%) were single. Regarding education, 1.6% held a high school diploma, 5.7% an associate degree, 59.0% an undergraduate degree, and 33.7% a graduate degree. Generational membership was distributed as 126 Generation X (13.8%, M age = 53.7, SD = 5.5), 222 Generation Y (24.2%, M age = 35.8, SD = 4.3), and 568 Generation Z (62.0%, M age = 21.4, SD = 2.3) respondents, for an overall mean age of 29.35 years (SD = 11.94, range 17–66) (Table 1).

### 2.4. Measures

Interpersonal communication quality was assessed with the seven-item Interpersonal Communication Scale (Campbell & Ataş Akdemir, 2016), which includes an External Perception subdimension (four items) and an Internal Sharing subdimension (three items). General phubbing and being phubbed were assessed with a combined 37-item instrument comprising the Generic Scale of Phubbing (GSP, 15 items) and the Generic Scale of Being Phubbed (GSBP, 22 items) (Chotpitayasunondh & Douglas, 2018a). The GSP comprises four validated subdimensions (Nomophobia, Interpersonal Conflict, Self-Isolation, and Problem Acknowledgment), and the GSBP comprises three (Perceived Norms, Feeling Ignored, and Interpersonal Conflict). These subdimensions were established in the scales’ original exploratory and confirmatory factor analyses (Chotpitayasunondh & Douglas, 2018a) and were retained without modification here. All items used a 5-point Likert format (1 = Strongly Disagree to 5 = Strongly Agree), and composite scores for each scale were computed as summed item totals rather than item means, following the scoring convention used in the scales’ original validation (Chotpitayasunondh & Douglas, 2018a); consequently, the theoretical range of each composite corresponds to its item count multiplied by 1 to 5 (e.g., 7–35 for communication quality, 15–75 for general phubbing, 22–110 for being phubbed).

Several GSP items index the Nomophobia subdimension, which captures phone-separation anxiety rather than the directly observable act of snubbing a conversational partner. This reflects the scale’s original multidimensional design, which included dependency-type items alongside behavioral items because earlier work had linked phubbing to broader patterns of phone, internet, and social media dependency. The four-subdimension structure was established through exploratory and confirmatory factor analyses in the scale’s original validation studies and was reproduced here (Section 3.1: acceptable to good CFA fit, all standardized loadings above 0.30). Thus, the composite is best read as a validated phubbing-proneness syndrome that spans dependency and behavior rather than as a narrow behavioral count of snubbing incidents.

### 2.5. Cross-Cultural Adaptation and Procedure

Because all three instruments were originally developed in English, they were adapted into Turkish using forward-and-back translation by independent bilingual translators, followed by expert review for wording. Semantic equivalence was checked by administering both language versions to the same bilingual respondents with an intervening interval; response consistency across administrations supported translation reliability. A pilot study (*n* = 172) was then conducted, and exploratory and confirmatory factor analyses on the pilot data indicated acceptable factor loadings and satisfactory sampling adequacy; the full item sets were retained unchanged for the main study. Reliability coefficients for the main sample are reported in Table 2; confirmatory factor analysis results establishing factorial validity for the main sample are reported in Section 3.1. Data collection took place on campus in 2025 via face-to-face administration to the final sample of 916 participants.

### 2.6. Ethical Considerations

The study was approved by the Atatürk University Social and Human Sciences Ethics Committee (session no. 19, decision no. 289, dated 5 November 2024; approval number E.88656144-000-2400365706). Because the questionnaire was distributed and completed anonymously in group settings, consent was obtained verbally rather than through a signed form: researchers read a standardized script informing potential participants of the study’s purpose, its voluntary and anonymous nature, and their right to decline or withdraw without consequence; participants who verbally agreed were then given the questionnaire, and completing it was treated as confirmation of consent. No personally identifying information was collected.

### 2.7. Data Analysis

Data were analyzed in SPSS 25.0 and AMOS 21. After screening for missing values, outliers, and coding errors, 916 valid cases were retained (missing data were minimal and handled through listwise deletion). One-way ANOVA was used to test generational differences in general phubbing, being phubbed, and communication quality (H1, H2, H7); simple linear regression was used to test the associations of general phubbing and being phubbed with communication quality (H3, H4); and hierarchical moderation regression (PROCESS version 4.2, Model 1, with Generation X coded as the reference category) was used to test whether generational membership moderated these associations (H5, H6).

For each ANOVA, the median-centered Levene’s test was used to assess homogeneity of variance; Bonferroni-corrected post hoc comparisons were used where this assumption held; and the Games–Howell procedure was used otherwise. For the H3 and H4 OLS regression models, assumptions were evaluated using standardized residual diagnostics. Residual histograms and normal P–P plots were inspected to assess the distribution of residuals, and plots of standardized residuals against standardized predicted values were examined to assess linearity and homoscedasticity. These graphical diagnostics were supplemented by formal Shapiro–Wilk tests of residual normality and Breusch–Pagan tests of heteroscedasticity. HC3 heteroscedasticity-consistent standard errors were additionally estimated as a robustness check to assess whether statistical inference for H3 and H4 was sensitive to departures from conventional OLS assumptions.

For the H5 and H6 moderation models, multicollinearity was assessed using variance inflation factors (VIFs). Because interaction terms constructed from uncentered continuous predictors can produce nonessential multicollinearity and inflate VIFs, the moderation models were additionally re-estimated with the focal continuous predictors mean-centered while retaining the same generational dummy coding. VIFs were then examined for these reparameterized models to evaluate whether the moderation results were affected by problematic multicollinearity.

Confirmatory factor analysis (AMOS 21) was additionally conducted on the main sample to verify the originally validated factor structure of each instrument. Within each multifactor CFA model, the latent factors were allowed to correlate. In the final measurement models, selected residual covariances were freely estimated only between indicators loading on the same latent factor; no residual covariances were specified between indicators loading on different factors. The significance threshold for all analyses was *p* < 0.05; because H1 through H7 test conceptually distinct relationships rather than repeated pairwise comparisons of a single outcome, no family-wise correction was applied across hypotheses, though within-test post hoc pairwise comparisons were corrected as described above.

As a check on whether the categorical generational variable adds explanatory value beyond chronological age, parallel regressions with age entered as a continuous variable were also estimated for general phubbing, being phubbed, and communication quality; these are reported alongside the generational analyses in Section 3.2. As an exploratory follow-up to the composite-level H1 analysis, separate one-way ANOVAs were conducted for the four GSP subdimensions to examine whether generational differences were also evident across the conceptually distinct components of the multidimensional scale. Subdimension scores were calculated directly from the constituent GSP items according to the validated four-factor scoring structure. These analyses were not specified a priori and are reported as supplementary rather than confirmatory tests in Section 3.2.2. A Bonferroni-adjusted significance threshold of *p* < 0.0125 was applied across these four exploratory subdimension tests. Generative AI (Claude Sonnet 5, Anthropic, San Francisco, CA, USA) was used to assist with supplementary statistical checks beyond the study’s primary analyses and the preparation of a figure; see the Acknowledgments section for details.

## 3. Results

### 3.1. Reliability of the Measures

Internal consistency was acceptable to excellent across all instruments (Table 2): the Interpersonal Communication Scale (α = 0.786), the Generic Scale of Phubbing (α = 0.880), and the Generic Scale of Being Phubbed (α = 0.922). Subdimension alphas ranged from 0.684 to 0.933.

Factorial validity was evaluated with confirmatory factor analysis (CFA; AMOS 21) on the main sample (*N* = 916), testing each instrument’s originally validated multi-factor structure. All three measurement models showed acceptable to good fit: the Interpersonal Communication Scale (two-factor, 7 items; χ^2^/df = 3.59, RMSEA = 0.053, SRMR = 0.027, CFI = 0.980, TLI = 0.965), the Generic Scale of Phubbing (GSP; four-factor, 15 items; χ^2^/df = 4.77, RMSEA = 0.064, SRMR = 0.025, CFI = 0.949, TLI = 0.934), and the Generic Scale of Being Phubbed (GSBP; three-factor, 22 items; χ^2^/df = 4.19, RMSEA = 0.059, SRMR = 0.054, CFI = 0.948, TLI = 0.940). The final CFA specification for the Interpersonal Communication Scale included one within-factor residual covariance between Items 2 and 3. For the GSP, two within-factor residual covariances were specified, between Items 5 and 6 and between Items 7 and 8, all within the Interpersonal Conflict factor. For the GSBP, five within-factor residual covariances were specified: between Items 1 and 2 and Items 3 and 4 within Perceived Norms; between Items 10 and 11 and Items 15 and 16 within Feeling Ignored; and between Items 18 and 19 within Interpersonal Conflict. No residual covariances were specified across latent factors. All freely estimated factor loadings were significant (*p* < 0.001), and standardized factor loadings exceeded the conventional 0.30 threshold (range 0.40–0.87 across the three instruments), supporting the structural validity of each measure in this sample rather than relying solely on the pilot-study factor analysis reported in Section 2.5. Item-level standardized and unstandardized factor loadings are reported in Appendix A.

### 3.2. H1 and H2: Generational Differences in General Phubbing and Being Phubbed

General phubbing increased stepwise across generations, F(2, 913) = 14.747, *p* < 0.001 (Generation X: M = 33.04; Generation Y: M = 36.58; Generation Z: M = 38.45), with Generation X scoring significantly lower than both Generation Y and Generation Z. This supports H1.

Being phubbed showed a different pattern: it did not differ significantly across generations, F(2, 913) = 0.031, *p* = 0.970 (Generation X: M = 69.66; Generation Y: M = 69.52; Generation Z: M = 69.33), in contrast to the significant generational difference observed for general phubbing. H2 was therefore also supported, with generational differences confined almost entirely to individuals’ own phubbing behavior rather than to their exposure to being phubbed.

Full ANOVA summary statistics for H1, H2, and H7, including sum of squares, mean squares, and effect sizes, are reported in Table 3.

#### 3.2.1. Generation Versus Chronological Age

Because generation and chronological age are severely collinear in this sample (Section 4.6), parallel regressions using age as a continuous predictor were run for each outcome to assess whether the categorical generational grouping adds anything beyond a simple age gradient. Age was significantly and negatively associated with general phubbing (β = −0.202, *p* < 0.001), explaining slightly more variance (R^2^ = 0.041) than the three-category generational grouping (R^2^ = 0.031), consistent with the stepwise increase already observed across Generations X, Y, and Z. Age was also significantly and positively associated with communication quality (β = 0.142, *p* < 0.001, R^2^ = 0.020), but the generational comparison did not follow a simple linear trend: Generation X and Generation Y did not differ from one another despite an average age gap of roughly 18 years, while Generation Z scored significantly lower than both (Section 3.5). The plateau between Generations X and Y followed by the lower Generation Z mean indicates that a simple linear age gradient does not fully reproduce the categorical pattern. However, this does not establish a cohort-specific mechanism: nonlinear age-related maturation, accumulated interpersonal experience, and cohort-related formative influences remain empirically inseparable in the present cross-sectional design. Generation is therefore retained as the categorical variable used to test H7, while the origin of the observed difference remains open to alternative explanations. Age was not significantly associated with being phubbed (β = 0.002, *p* = 0.948), mirroring the null generational difference reported above and indicating that the H2 null result is not an artifact of using generational categories instead of continuous age.

#### 3.2.2. Exploratory GSP Subdimension Analysis

Because the GSP composite aggregates subdimensions with different conceptual content (Section 2.4), an exploratory analysis examined whether generational differences were also evident across its four subdimensions. This analysis was not specified a priori and is reported as a supplementary check rather than as a confirmatory test of H1. Subdimension scores were calculated directly from the constituent GSP items according to the validated four-factor scoring structure. All four subdimensions showed significant generational differences after application of the Bonferroni-adjusted threshold of *p* < 0.0125: Nomophobia (Generation X: M = 11.19; Generation Y: M = 12.52; Generation Z: M = 12.38), F(2, 913) = 4.954, *p* = 0.007, η^2^ = 0.011; Interpersonal Conflict (X: M = 7.56; Y: M = 8.03; Z: M = 8.70), F(2, 913) = 7.512, *p* < 0.001, η^2^ = 0.016; Self-Isolation (X: M = 6.91; Y: M = 7.45; Z: M = 8.57), F(2, 913) = 17.234, *p* < 0.001, η^2^ = 0.036; and Problem Acknowledgment (X: M = 7.37; Y: M = 8.59; Z: M = 8.80), F(2, 913) = 12.932, *p* < 0.001, η^2^ = 0.028. The median-centered Levene’s test indicated heterogeneity of variance for Self-Isolation (W = 6.46, *p* = 0.002); a Welch-corrected robustness check confirmed the same conclusion, F(2, 336.3) = 20.62, *p* < 0.001. Variances were homogeneous for the remaining three subdimensions. The smallest generational effect was observed for Nomophobia, whereas the largest was observed for Self-Isolation. Unlike the composite H1 pattern, the descriptive Nomophobia means were not monotonic across generations, with Generation Y showing a slightly higher mean than Generation Z.

### 3.3. H3 and H4: Associations of General Phubbing and Being Phubbed with Communication Quality

General phubbing was significantly and negatively associated with communication quality, F(1, 914) = 20.710, *p* < 0.001, β = −0.149, B = −0.063, adjusted R^2^ = 0.021, supporting H3. Being phubbed showed the opposite pattern: it was significantly, though weakly, positively associated with communication quality, F(1, 914) = 5.992, *p* = 0.015, β = 0.081, B = 0.024, adjusted R^2^ = 0.005. H4 was not supported, since the hypothesized negative association was not observed; this divergence is examined in Section 4.3.

Residual diagnostics indicated departures from normality for both OLS models (H3: Shapiro–Wilk W = 0.961, *p* < 0.001; H4: W = 0.961, *p* < 0.001). Breusch–Pagan tests provided no statistically significant evidence of heteroscedasticity for either H3 (LM = 0.84, *p* = 0.360) or H4 (LM = 3.73, *p* = 0.054). As a conservative robustness check in light of these residual distributional departures, both models were re-estimated using HC3 heteroscedasticity-consistent standard errors. The substantive conclusions were unchanged: the negative association for H3 remained significant (*p* < 0.001), and the unexpected positive association for H4 also remained significant (*p* = 0.031).

#### Post Hoc Disaggregation of the GSBP Composite

To explore whether the unexpected positive composite association observed for H4 might mask opposing subdimension-level relationships, communication quality was regressed simultaneously on the three GSBP subdimensions. The model was significant, F(3, 912) = 20.22, *p* < 0.001, adjusted R^2^ = 0.059. Perceived Norms showed a significant positive association with communication quality (*p* < 0.001), whereas Feeling Ignored showed a negative but non-significant association (*p* = 0.067), and Interpersonal Conflict likewise showed a negative, non-significant association (*p* = 0.185). These results are consistent with the composite-masking explanation offered below (Section 4.3): the positive composite association appears to be concentrated primarily in the Perceived Norms subdimension, while the more affectively loaded subdimensions showed associations in the negative direction. This post hoc analysis was not pre-registered and should be interpreted as hypothesis-generating rather than confirmatory.

### 3.4. H5 and H6: Generational Moderation

Generational membership did not moderate the association between general phubbing and communication quality, supporting H5. The overall model was significant, F(5, 910) = 9.79, *p* < 0.001, R^2^ = 0.051, but neither interaction term was statistically significant (ΔR^2^ = 0.0047, *p* = 0.107; general phubbing × Generation Y, *p* = 0.826; general phubbing × Generation Z, *p* = 0.127).

Full hierarchical regression results for H5, including unstandardized coefficients, standard errors, and confidence intervals for each step, are reported in Table 4.

The same pattern held for being phubbed, supporting H6. The overall model was again significant, F(5, 910) = 7.23, *p* < 0.001, R^2^ = 0.0382, yet neither interaction term was statistically significant (being phubbed × Generation Y, *p* = 0.224; being phubbed × Generation Z, *p* = 0.600; ΔR^2^ = 0.0019, F(2, 910) = 0.92, *p* = 0.399).

Multicollinearity diagnostics for the moderation models were examined after mean-centering the focal continuous predictors while retaining the original generational dummy coding. The maximum VIF was 8.84 for H5 and 8.25 for H6. Mean-centering did not alter the interaction coefficients, their standard errors, or the corresponding significance tests; therefore, the substantive conclusions for H5 and H6 remained unchanged.

Full hierarchical regression results for H6, including unstandardized coefficients, standard errors, and confidence intervals for each step, are reported in Table 5.

### 3.5. H7: Generational Differences in Communication Quality

Communication quality differed significantly across generations, supporting H7: F(2, 913) = 14.106, *p* < 0.001. Levene’s test indicated heterogeneity of variance across the three generational groups for this variable (W = 12.32, *p* < 0.001); post hoc comparisons therefore used the Games–Howell procedure rather than Bonferroni, following the variance-dependent post hoc selection rule described in Section 2.7. These comparisons showed Generation Z (M = 27.24) scoring significantly lower than both Generation X (M = 28.60, *p* = 0.004) and Generation Y (M = 28.92, *p* < 0.001), which did not differ from one another (*p* = 0.744). As a robustness check on the unequal-variance assumption, the omnibus test was re-estimated with Welch’s F, which confirmed the same conclusion, F(2, 327.5) = 16.76, *p* < 0.001. For H1 and H2, Levene’s test indicated homogeneous variances across groups (general phubbing: W = 1.82, *p* = 0.162; being phubbed: W = 0.24, *p* = 0.785), so the standard ANOVA and Bonferroni-corrected post hoc comparisons reported above are appropriate as originally presented.

As a sensitivity check on the generational comparisons underlying H1 and H7, both ANOVAs were re-estimated after excluding the 14 participants aged above 60 from the Generation X subsample (*n* = 902). The pattern of results was unchanged: general phubbing still differed significantly by generation, F(2, 899) = 11.843, *p* < 0.001 (Generation X: M = 33.45; Generation Y: M = 36.58; Generation Z: M = 38.45), and communication quality still differed significantly by generation, F(2, 899) = 12.834, *p* < 0.001 (Generation X: M = 28.36; Generation Y: M = 28.92; Generation Z: M = 27.24). The inclusion of participants above the conventional 60-year cutoff in Generation X therefore did not drive either result.

### 3.6. Summary of Hypothesis Testing

A summary of the hypothesis-testing results is presented in Table 6.

## 4. Discussion

### 4.1. Generational Patterns: Phubbing Is Generational, Being Phubbed Is Not

The central finding in these data is an asymmetry between the two phubbing constructs. General phubbing rose steadily from Generation X to Z (H1), consistent with the hypothesized cohort pattern. Because generational membership is not a direct measure of individual digital exposure or technological habituation, this result should be interpreted as a difference between the operational birth-year groups rather than as evidence that “digital-native” status itself produced higher phubbing. Being phubbed, by contrast, was essentially identical across the three cohorts (H2). Taken together, these results support the normalization account outlined in the introduction more precisely than a simple, generation-uniform prediction would: generational membership is associated with differences in how often a person turns to a phone during interaction, but not with how often that person perceives being on the receiving end of someone else’s phone use (Chotpitayasunondh & Douglas, 2016). Because being phubbed depends on a partner’s behavior and the surrounding communicative context rather than the respondent’s own cohort, its near-uniform distribution across generations suggests that phubbing has become embedded as a structural feature of contact between generations, not solely a within-generation phenomenon.

An important qualification concerns what the GSP composite represents in this comparison. As described in Section 2.4, the GSP does not constitute a narrow behavioral count of observable snubbing episodes; it combines Interpersonal Conflict and Problem Acknowledgment with Self-Isolation and Nomophobia, the latter indexing anxiety associated with separation from one’s phone rather than the directly observable act of snubbing a partner (Chotpitayasunondh & Douglas, 2018a). Accordingly, the generational difference observed for H1 should be interpreted as a difference in the broader GSP-defined phubbing-proneness construct rather than as unequivocal evidence that the cohorts differ to the same extent in observable conversational snubbing itself. An exploratory subdimension-level analysis (Section 3.2.2) provides some additional context: generational differences remained significant across all four GSP subdimensions after correction for multiple testing, while the effect size was smallest for Nomophobia (η^2^ = 0.011) and largest for Self-Isolation (η^2^ = 0.036). This pattern suggests that the H1 composite difference is unlikely to be attributable solely to the Nomophobia component. However, because these analyses were exploratory and separate subdimension-level ANOVAs do not decompose the composite effect into unique contributions of each component, the present data cannot establish how much of the overall H1 effect is specifically behavioral rather than dependency-related.

### 4.2. Associations with Communication Quality: A Supported and an Unsupported Hypothesis

General phubbing showed the hypothesized negative association with communication quality (H3). This aligns with both Media Richness Theory (Daft & Lengel, 1986) and the interaction involvement framework (Cegala, 1981), and it converges with Dwyer et al.’s (2018) restaurant field experiment, in which diners who kept their phones on the table reported less enjoyment of the meal than those who put them away. The authors traced this effect to distraction rather than to any single act of phone use. The present association is consistent with that logic at the level of habitual phone-oriented behavior, and it converges with survey evidence that heavier phubbers report weaker relational outcomes with friends and romantic partners alike (Roberts & David, 2016; Sun & Samp, 2022). The effect size found here was modest, consistent with a broader literature that treats phubbing as one correlate of communication quality among many rather than a dominant one (Liu et al., 2010).

### 4.3. The Unexpected Positive Association of Being Phubbed

Being phubbed did not show the hypothesized negative association; instead, it showed a small positive association with communication quality, and H4 was not supported. This result contrasts with the dyadic diary study by Carnelley et al. (2023) of romantic couples, which distinguished perceived partner phubbing from partners’ own self-reported, or enacted, phubbing and found that only the perceived measure predicted lower relationship quality, while enacted phubbing showed no relationship to it at all. The GSBP used here asks respondents to report their own perception of being phubbed, which is conceptually closer to Carnelley et al.’s perceived-phubbing measure than to their enacted-phubbing measure, for which no association with relationship quality was observed, so the direction of the present finding, not merely its size, is difficult to reconcile with that study at the composite score level. Disaggregating the GSBP helps clarify this discrepancy. Because the composite aggregates a Perceived Norms subdimension (“others seem to check their phones”) with a more affectively loaded Feeling Ignored subdimension, a positive relationship at the composite level could mask opposing relationships at the subdimension level; a norm-perception item does not necessarily carry the same relational weight as a direct feeling of being ignored. A post hoc analysis of this possibility (see the subsection “Post Hoc Disaggregation of the GSBP Composite”) is consistent with this account: Perceived Norms showed a significant positive association with communication quality, while Feeling Ignored showed a negative association in the direction predicted by Carnelley et al.’s perceived-phubbing findings but did not reach statistical significance, and Interpersonal Conflict likewise showed a negative, non-significant association. Because this disaggregation was not specified in advance, it should be treated as an exploratory indication rather than as confirmation of the composite-masking account, and future work should pre-register subdimension-level hypotheses for the GSBP directly, ideally alongside a dyadic design like Carnelley et al.’s that can separate the phubber’s and phubbee’s reports.

Two further explanations remain plausible and are not exclusive of this one. Respondents who report high overall communication quality may also be more socially attentive observers who are simply more likely to notice and report others’ phone use in general, without that noticing translating into felt neglect. Alternatively, being embedded in relationships or settings with frequent mutual phone co-presence, of the kind reported in workplace and supervisor-subordinate contexts (Roberts & David, 2020), may reflect relationships that are already comfortable enough to tolerate device use without a loss of perceived quality, a possibility that Dwyer et al.’s (2018) observation that phone-related distraction is most pronounced when phone use is mismatched between interaction partners would also support. Given the very small composite-level effect size (β = 0.081, adjusted R^2^ = 0.005), this finding is best treated as a divergence worth further, targeted investigation rather than as evidence against the broader literature on the relational costs of being phubbed.

### 4.4. Generational Invariance of the Observed Associations

Neither the negative association between general phubbing and communication quality (H5) nor the weak positive association between being phubbed and communication quality (H6) was moderated by generational membership, as hypothesized. This pattern indicates that the observed associations between the two phubbing measures and communication quality did not differ significantly across the three generational groups, even though general phubbing levels differed across generations (H1). The findings are therefore consistent with, but do not establish, the possibility that attentional processes relevant to face-to-face interaction operate similarly across generational groups (Cegala, 1981; Daft & Lengel, 1986).

### 4.5. Generation Z and Communication Quality

Generation Z reported the lowest communication quality among the three cohorts (H7). This result held even after excluding the 14 participants classified as Generation X above the conventional 60-year cutoff (Section 3.5), reducing the likelihood that the finding is an artifact of that classification. The statistical difference itself is clear, but its underlying explanation is less certain. Importantly, Generation Z membership in the present study reflects an operational demographic classification based on birth year; it is not a direct measure of an individual’s digital exposure, smartphone use, technological competence, or digital dependence. Although cohort theory and the digital-native framework motivated the original expectation, the present cross-sectional design cannot determine whether Generation Z’s lower self-reported communication quality reflects cohort-specific technological formation, chronological age, maturation, accumulated interpersonal experience, or some combination of these influences. The supplementary age analysis showed that chronological age was positively associated with communication quality, while Generations X and Y did not differ despite a substantial difference in mean age. This suggests that a simple linear age gradient does not fully account for the observed pattern, but it does not rule out nonlinear maturation or life-experience effects. This distinction is consistent with critical accounts of the digital-native construct, which caution against attributing heterogeneous cohort differences to a single technological mechanism (Bayne & Ross, 2007; Bennett et al., 2008; Brown & Czerniewicz, 2010). Accordingly, the present data support the limited conclusion that Generation Z reported lower communication quality than the two older cohorts in this sample; they do not establish digital exposure or attentional fragmentation as the cause of that difference. Generational categories are therefore treated here as analytical classifications intended to capture possible differences associated with shared formative periods, not as deterministic descriptions of individual communicative ability. The observed cohort-level averages should not be interpreted as implying the superiority or inferiority of any generation, nor should generational membership be used to infer an individual’s communication competence. From this perspective, generational differences are more appropriately understood as possible differences in the baseline of behavioral norms shaped by distinct formative environments, including the extent to which smartphone presence is taken for granted in everyday interaction, rather than as differences in underlying communicative ability.

This distinction is important because interpreting a group-level difference as an inherent characteristic of individual Generation Z members could reproduce the very stereotyping problem associated with the digital-native/immigrant binary. The present findings should therefore not be used to infer that younger individuals are intrinsically poorer communicators or that older individuals are inherently less digitally capable; such conclusions would convert probabilistic cohort-level patterns into fixed judgments about individuals and would disregard substantial within-generation heterogeneity.

This caution also has practical implications for organizational and educational settings. A lower group mean in communication quality should not be converted into a proxy for individual communicative competence, particularly where communication style contributes to judgments of participation, professionalism, or performance. Research on workplace ageism shows that age categories and associated stereotypes can enter performance appraisal and other evaluative processes, and recent work demonstrates that such bias can also be directed toward younger workers in the form of workplace “youngism” (Previtali & Spedale, 2021; Schmitz et al., 2025). The present finding should therefore not be used to justify evaluative disadvantage for members of Generation Z. In employment contexts, this risk is particularly relevant to hiring interviews and performance reviews: treating traditional face-to-face fluency as the primary or sole indicator of communication competence could unfairly disadvantage younger applicants or employees whose effective communication may also be expressed through other modalities. Nor does it imply that face-to-face interaction should constitute the sole normative standard of effective communication. An accommodation-oriented approach would instead distinguish communicative effectiveness from communication modality, allowing context-appropriate use of face-to-face, text-based, synchronous, and asynchronous channels while evaluating individuals on task-relevant communicative outcomes rather than presumed generational characteristics. Accordingly, modality-independent evaluation rubrics could assess communication competence through criteria such as successful completion of collaborative tasks in asynchronous text-based settings, clarity and accuracy in digital information sharing, responsiveness to communication demands, and effective adaptation across communication channels. This position is consistent with Communication Accommodation Theory, which emphasizes adaptive adjustment to interlocutors and communicative contexts, including computer-mediated interaction (Giles et al., 2023).

### 4.6. Limitations

The most consequential limitation concerns data that were never collected: occupational or student status was not recorded in this study. Because the sampled population combined students with academic and administrative staff, and these groups plausibly differ in age and therefore in generational membership, the present data cannot rule out the possibility that the generational patterns reported here partly reflect uncontrolled differences in occupational context rather than generational membership alone. Because occupational or student status was not recorded in the questionnaire and no recruitment or administration records were retained from which this information could be reconstructed retrospectively, the exact distribution of students, academic staff, and administrative staff within each generational cohort cannot be determined. Accordingly, potential differences associated with student–staff roles, institutional context, or status-related interaction dynamics cannot be disentangled from the observed generational patterns. For H7 in particular, Generation Z’s lower self-reported communication-quality scores may therefore partly reflect student–staff role differences and associated institutional power gradients rather than cohort membership per se; the present data cannot distinguish between these alternative explanations. This unmeasured status confounding is therefore treated as a major threat to the internal validity of generation-based comparisons and inferences, particularly the H1 and H7 group differences, rather than as a routine limitation. Future replications should record and control for occupational or employment status explicitly.

A related constraint concerns measurement scope rather than measurement quality. As noted in Section 2.4, the GSP and GSBP composites aggregate psychometrically distinct subdimensions, including constructs such as nomophobia that index general phone dependency alongside the directly observable act of snubbing a partner. Both instruments showed strong internal consistency (α = 0.880 and 0.922) and acceptable to good CFA fit in the present sample (Section 3.1), consistent with their original validation (Chotpitayasunondh & Douglas, 2018a). Thus, the composites can be treated as reliable and structurally valid measures of the broader constructs they were designed to capture. However, this multidimensional design limits precision at the behavioral level: because “general phubbing,” as operationalized here, spans dependency and behavior, the exploratory GSP subdimension analysis reported in Section 3.2.2 provides additional context but does not isolate the unique behavioral contribution of each component. Future studies designed specifically to distinguish observable snubbing from broader phubbing-proneness should therefore pre-register and test subdimension-level hypotheses.

A related interpretive limitation concerns H4. The composite-masking explanation offered for the unexpected positive association between being phubbed and communication quality relies on a post hoc GSBP subdimension analysis that was not specified a priori. Although the observed pattern is consistent with Perceived Norms contributing disproportionately to the positive composite association, the analysis cannot establish this as the underlying mechanism. The interpretation should therefore be regarded as hypothesis-generating until it is replicated in confirmatory, preferably pre-registered, subdimension-level analyses.

Several additional limitations should also be noted. The sample was drawn from a single Turkish public university using nonprobability convenience sampling, limiting generalizability to other institutional, regional, or cultural contexts. Given documented cross-cultural variation in how co-present phone use is interpreted (Büttner et al., 2025), replication elsewhere is particularly warranted. The cross-sectional design precludes causal inference in either direction. All measures relied on self-report and may be subject to social desirability or recall bias. Because all focal variables were assessed using self-report measures within the same questionnaire, shared method variance and response-style tendencies may also have inflated or otherwise distorted some of the observed associations. In addition, generational differences in self-perception or reporting style cannot be ruled out as a contributor to the between-group differences observed here. The modest variance explained by the regression models (adjusted R^2^ = 0.005 to 0.021) indicates that much of the variance in communication quality remains unexplained by phubbing and being phubbed, pointing to other relevant factors not modeled here. A further consequence of the cross-sectional design is that generational membership and chronological age are severely collinear in this sample (r = −0.96; variance inflation factors of 7 to 23 when both are entered as predictors), because generation was itself assigned from birth year. This makes it statistically impossible, with the present data, to attribute the reported generational differences (H1, H7) to cohort-specific formative experience as opposed to age itself. For H7 specifically, age-related maturation and accumulated interpersonal experience therefore remain plausible alternative explanations for Generation Z’s lower self-reported communication-quality scores. The two accounts cannot be separated within a single cross-sectional sample, and only a longitudinal or time-lag design that follows or repeatedly samples the same birth cohorts over time could adjudicate between them. The supplementary age-based analysis in Section 3.2.1 partly mitigates this concern for general phubbing and being phubbed, where age alone reproduces the generational finding, but the non-monotonic relationship found for communication quality is not fully reducible to a linear age effect and should be interpreted with this collinearity in mind.

### 4.7. Future Research

Future studies should replicate this model with occupational status recorded and controlled, in more diverse and representative samples spanning multiple institutions and cultural contexts, and with longitudinal or time-lag designs capable of separating cohort-related technological formation from age-related maturation and accumulated interpersonal experience. Pre-registering and testing GSBP subdimension-level hypotheses, particularly for Perceived Norms, Feeling Ignored, and Interpersonal Conflict, would help determine whether the exploratory pattern observed for H4 replicates in confirmatory analyses. Additional moderators worth testing include relationship type between interaction partners, smartphone dependency, and specific communication contexts (e.g., work versus leisure), and the generational comparison could usefully be extended to Generation Alpha as this cohort enters early adulthood.

## 5. Conclusions

This study found that general phubbing, but not being phubbed, varied systematically across Generations X, Y, and Z, with Generation Z reporting the highest general phubbing scores. General phubbing was consistently and negatively associated with communication quality, whereas being phubbed showed a small, unexpected positive association; neither relationship was moderated by generational membership, indicating that the observed associations did not differ significantly across the three generational groups. Generation Z also reported the lowest overall communication quality, a result that proved robust to classifying older participants within Generation X. Importantly, this group-level difference should not be interpreted as evidence of an inherent communicative deficit in Generation Z or used as a proxy for individual competence in educational or workplace evaluations. Where communication style contributes to judgments of participation, professionalism, or performance, cohort-based expectations may introduce age-based evaluative bias. A more appropriate practical implication is to support context-sensitive communication practices in which face-to-face interaction is one option among several, including text-based and asynchronous formats, and to evaluate individuals according to task-relevant communicative outcomes rather than presumed generational traits. Taken together, these findings identify a generational asymmetry in the observed pattern of phubbing and communication quality, but they do not establish that digital exposure or attentional fragmentation caused the lower communication-quality scores observed in Generation Z. Age-related maturation, accumulated interpersonal experience, cohort-specific technological formation, and other unmeasured influences remain viable explanations that cannot be disentangled within the present cross-sectional design. At the same time, exposure to being phubbed appears to have become broadly shared across generations, consistent with the normalization account advanced in this study.

## Figures and Tables

**Table 1:** Sample characteristics (*N* = 916). Occupational/employment status was not recorded; see Section 4.6 for the resulting limitation.

Variable	Category	*n*	%
Gender	Male	427	46.6
	Female	489	53.4
Marital status	Married	240	26.2
	Single	676	73.8
Education	High school	15	1.6
	Associate degree	52	5.7
	Undergraduate	540	59.0
	Graduate	309	33.7
Generation	X (M age = 53.7, SD = 5.5)	126	13.8
	Y (M age = 35.8, SD = 4.3)	222	24.2
	Z (M age = 21.4, SD = 2.3)	568	62.0

**Table 2:** Reliability coefficients for the study measures.

Scale	Subdimension	Cronbach’s α
Interpersonal Communication Scale	Overall	0.786
	External Perception	0.719
	Internal Sharing	0.700
Generic Scale of Phubbing (GSP)	Overall	0.880
	Nomophobia	0.864
	Interpersonal Conflict	0.774
	Self-Isolation	0.848
	Problem Acknowledgment	0.684
Generic Scale of Being Phubbed (GSBP)	Overall	0.922
	Perceived Norms	0.878
	Feeling Ignored	0.933
	Interpersonal Conflict	0.874

**Table 3:** ANOVA summary for generational comparisons (H1, H2, H7).

Source	SS	df	MS	F	*p*	η^2^
H1—General phubbing (between groups)	3149.38	2	1574.69	14.747	<0.001	0.031
H1—(within groups)	97,489.36	913	106.78			
H2—Being phubbed (between groups)	13.81	2	6.91	0.031	0.970	0.0001
H2—(within groups)	206,493.53	913	226.17			
H7—Communication quality (between groups)	533.08	2	266.54	14.106	<0.001	0.030
H7—(within groups)	17,251.68	913	18.90			

*Note.* For H7, variances were heterogeneous across groups (Levene’s W = 12.32, *p* < 0.001); a Welch-corrected robustness check is reported in Section 3.5.

**Table 4:** Hierarchical regression of communication quality on general phubbing and generational membership (H5).

Predictor	B	SE	95% CI	*p*
Step 1				
Constant	30.409	0.597	[29.238, 31.579]	<0.001
General phubbing	−0.055	0.014	[−0.082, −0.028]	<0.001
Generation Y	0.509	0.484	[−0.440, 1.458]	0.293
Generation Z	−1.063	0.431	[−1.909, −0.217]	0.014
Step 2				
Constant	28.949	1.388	[26.225, 31.672]	<0.001
General phubbing	−0.011	0.040	[−0.090, 0.069]	0.796
Generation Y	0.743	1.720	[−2.633, 4.119]	0.666
Generation Z	1.281	1.554	[−1.769, 4.331]	0.410
Phubbing × Generation Y	−0.011	0.048	[−0.106, 0.084]	0.826
Phubbing × Generation Z	−0.067	0.044	[−0.154, 0.019]	0.127

*Note.* Generation X is the reference category. Step 1 R^2^ = 0.046; Step 2 R^2^ = 0.051; ΔR^2^ = 0.0047, F-change(2, 910) = 2.245, *p* = 0.107.

**Table 5:** Hierarchical regression of communication quality on being phubbed and generational membership (H6).

Predictor	B	SE	95% CI	*p*
Step 1				
Constant	26.979	0.769	[25.471, 28.488]	<0.001
Being phubbed	0.023	0.009	[0.005, 0.042]	0.015
Generation Y	0.319	0.484	[−0.630, 1.268]	0.510
Generation Z	−1.351	0.427	[−2.189, −0.513]	0.002
Step 2				
Constant	25.577	1.947	[21.756, 29.398]	<0.001
Being phubbed	0.043	0.027	[−0.010, 0.097]	0.113
Generation Y	3.142	2.368	[−1.505, 7.789]	0.185
Generation Z	−0.254	2.127	[−4.429, 3.921]	0.905
Being phubbed × Generation Y	−0.041	0.033	[−0.106, 0.025]	0.224
Being phubbed × Generation Z	−0.016	0.030	[−0.075, 0.043]	0.600

*Note.* Generation X is the reference category. Step 1 R^2^ = 0.036; Step 2 R^2^ = 0.038; ΔR^2^ = 0.0019, F-change(2, 910) = 0.920, *p* = 0.399.

**Table 6:** Summary of hypothesis testing results.

Hypothesis	Statistical Result	Decision
H1 (general phubbing increases X → Y→ Z)	F(2, 913) = 14.747, *p* < 0.001	Supported
H2 (generational gap smaller for being phubbed)	F(2, 913) = 0.031, *p* = 0.970	Supported
H3 (general phubbing–comm. quality, negative association)	F = 20.710, *p* < 0.001, β = −0.149	Supported
H4 (being phubbed–comm. quality, negative association)	F = 5.992, *p* = 0.015, β = 0.081	Not supported (opposite direction)
H5 (no generational moderation, general phubbing)	ΔR^2^ = 0.0047, *p* = 0.107	Supported
H6 (no generational moderation, being phubbed)	ΔR^2^ = 0.0019, *p* = 0.399	Supported
H7 (Gen Z lowest comm. quality)	F(2, 913) = 14.106, *p* < 0.001	Supported

## Data Availability

The data presented in this study are openly available in the Supplementary Materials associated with this article.

## Supplementary Materials

The following supporting information can be downloaded at: https://www.mdpi.com/article/10.3390/bs16091536/s1, the updated anonymized dataset and the variable codebook.

## Appendix A

Table A1, Table A2, and Table A3 report the standardized and unstandardized item-level factor loadings from the confirmatory factor analyses summarized in Section 3.1, for the Interpersonal Communication Scale, the Generic Scale of Phubbing (GSP), and the Generic Scale of Being Phubbed (GSBP), respectively.

**Table A1:** CFA item loadings—Interpersonal Communication Scale (7 items).

Item	Standardized Loading	Unstandardized Loading	SE	CR	*p*
Item 1	0.659	1.000	—	—	—
Item 2	0.639	1.039	0.073	14.138	<0.001
Item 3	0.511	0.860	0.073	11.802	<0.001
Item 4	0.617	0.952	0.068	14.084	<0.001
Item 5	0.547	1.000	—	—	—
Item 6	0.811	1.324	0.093	14.194	<0.001
Item 7	0.649	0.981	0.073	13.379	<0.001

*Note.* CR = critical ratio (t-value); item numbers correspond to the original item order in the survey instrument.

**Table A2:** CFA item loadings—Generic Scale of Phubbing (GSP, 15 items).

Item	Standardized Loading	Unstandardized Loading	SE	CR	*p*
Item 1	0.814	1.000	—	—	—
Item 2	0.872	1.075	0.038	28.262	<0.001
Item 3	0.713	0.818	0.036	22.635	<0.001
Item 4	0.728	0.887	0.038	23.233	<0.001
Item 5	0.624	1.000	—	—	—
Item 6	0.673	1.114	0.062	18.034	<0.001
Item 7	0.661	0.986	0.067	14.653	<0.001
Item 8	0.626	0.885	0.063	14.075	<0.001
Item 9	0.704	1.000	—	—	—
Item 10	0.832	1.198	0.053	22.596	<0.001
Item 11	0.833	1.191	0.053	22.606	<0.001
Item 12	0.704	1.057	0.054	19.489	<0.001
Item 13	0.735	1.000	—	—	—
Item 14	0.444	0.628	0.054	11.648	<0.001
Item 15	0.798	1.155	0.067	17.268	<0.001

**Table A3:** CFA item loadings—Generic Scale of Being Phubbed (GSBP, 22 items).

Item	Standardized Loading	Unstandardized Loading	SE	CR	*p*
Item 1	0.398	1.000	—	—	—
Item 2	0.438	1.064	0.076	13.969	<0.001
Item 3	0.569	1.277	0.122	10.444	<0.001
Item 4	0.725	1.659	0.146	11.401	<0.001
Item 5	0.724	1.710	0.150	11.404	<0.001
Item 6	0.781	1.774	0.152	11.650	<0.001
Item 7	0.795	1.859	0.159	11.705	<0.001
Item 8	0.695	1.600	0.142	11.259	<0.001
Item 9	0.737	1.748	0.153	11.463	<0.001
Item 10	0.660	1.000	—	—	—
Item 11	0.773	1.177	0.044	27.067	<0.001
Item 12	0.815	1.325	0.061	21.568	<0.001
Item 13	0.834	1.322	0.060	21.968	<0.001
Item 14	0.848	1.371	0.062	22.248	<0.001
Item 15	0.820	1.305	0.060	21.631	<0.001
Item 16	0.802	1.274	0.060	21.234	<0.001
Item 17	0.775	1.209	0.058	20.679	<0.001
Item 18	0.598	1.000	—	—	—
Item 19	0.757	1.252	0.060	20.805	<0.001
Item 20	0.781	1.330	0.075	17.824	<0.001
Item 21	0.823	1.392	0.076	18.381	<0.001
Item 22	0.808	1.391	0.077	18.184	<0.001
