# Supplementary material for: Phubbing, Being Phubbed, and Interpersonal Communication Quality Across Generations X, Y, and Z: Evidence of a Generational Asymmetry
Source: Behav Sci (Basel). 2026 Aug 31;16(9):1536. doi: 10.3390/bs16091536 (PMC13603854; doi:10.3390/bs16091536)
Supplement: Supplementary file 1 [file behavsci-16-01536-s001.zip › variable codebook.pdf]

Variable Codebook

This document provides definitions and coding details for variables included in the dataset.

Demographic variables

Gender
0 = Male
1 = Female

Marital status
0 = Single
1 = Married

Education level
1 = High school
2 = Associate degree
3 = Undergraduate
4 = Postgraduate

Generation
1 = Generation X (1965–1980)
2 = Generation Y (1981–1996)
3 = Generation Z (1997–2012)

Age
Continuous variable in years.

Study variables

Perceived interpersonal communication quality
Scale: Interpersonal Communication Scale
Items: 7
Range: 7–35 (sum of 7 items, each scored 1–5)
Higher scores indicate higher perceived communication quality.
Total score computed as sum of items (not mean), following the scale's original scoring convention.

Phubbing
Scale: Generic Scale of Phubbing (GSP)
Items: 15
Range: 15–75 (sum of 15 items, each scored 1–5)
Higher scores indicate higher phubbing behaviour.
Total score computed as sum of items (not mean), following the scale's original scoring convention.

Being phubbed
Scale: Generic Scale of Being Phubbed (GSBP)
Items: 22
Range: 22–110 (sum of 22 items, each scored 1–5)

Higher scores indicate higher exposure to others’ phone use.
Total score computed as sum of items (not mean), following the scale's original scoring convention.

Missing data

Missing values were minimal and handled using listwise deletion in analyses.
No cases were removed except for incomplete responses.
